# Joseph Francis Domin's (1754-1819) first description of human resuscitation with electricity one hundred years before clinical defibrillation

**DOI:** 10.3325/cmj.2021.62.192

**Published:** 2021-04

**Authors:** Dubravko Habek, Jasna Čerkez, Habek, Mirjana Matijević, Sokol, Ivana Horbec

**Affiliations:** 1 Catholic University of Croatia, Zagreb, Croatia; 2 Sveti Duh Clinical Hospital, Zagreb, Croatia; 3 Croatian Academy of Medical Sciences, Zagreb, Croatia; 4 Croatian Institute of History, Zagreb, Croatia

Numerous iatrophysicists and iatrochemists were experimenting with the effects of external forces on morphological and physiological processes, including the influence of electricity on animal and human organisms. They specifically investigated the effects on muscular activity, circulation, and nervous system, and their works served as a basis for the subsequent use of electricity in diagnosis and treatment, as well as in cardiocirculatory resuscitation (1). Valuable historical review papers have been published on the use of electrotherapy in the treatment of cardiac arrhythmia, cardiac arrest, and sudden death (2-6).

Noticeable scientific and professional work in medical electrotherapy was performed by Joseph Francis Domin (1754–1819). Domin was a scientist of Croatian origin, who lived in the Habsburg Monarchy in the period of Enlightenment and a rapid development of natural science. As a philosopher, theologist, and natural scientist, Domin was appointed professor of theoretical and experimental physics, mechanics, and chemistry at the Royal Academies in Győr and Pecs, as well as professor of physics at the Faculty of Philosophy of the University of Pest, where he also served as Dean. Domin wrote several works in Latin on the use of electricity in medical treatments based on his own experiences. In these works, he described the beneficial effects of electricity on the treatment of paresis, rheumatism, headache, epilepsy, podagra, febrile conditions in children and adults, hearing loss, neuralgia, and resuscitation after asphyxia (7-13).

Here, we present our English translation of Domin’s description of simultaneous resuscitation procedure with static electricity and oxygenation. This was the first text on that topic in the scientific literature of that time. This text allows new insights into the treatment of cardiac arrest at the end of the 18th century – one century before the first proven use of electrotherapy in clinical human resuscitation.

## J. F. DOMIN AND THE ELECTRICAL RESUSCITATION IN CARDIAC ARREST

In his work, Domin described how static electricity accelerated the circulation, resulting in the acceleration of the pulse (tachycardia), fever, perspiration, secretion of the glands, and maintaining the density to the appropriate extent, reducing obstruction in small blood vessels, as well as facilitating secretion, opening obstructed natural openings, loosening myofibrils, irritating the nerve fibers, and thus reviving the apparently dead (10). In his latest and the most comprehensive work from 1796, *Ars Electricitatem Aegris Tuto Adhibendi, Cum Propriis, Tum Aliorum Virorum Celeberrimorum Experimentatis Innixa,* Domin exhibits basic and advanced knowledge of the physics of electricity and its use in medical treatments, thus heralding the dawn of biophysics. In the chapter CXV, he describes the resuscitation procedure by applying an electrical shock to an apparently dead person (*mors apparens*). Although Domin was not a physician, he performed the resuscitation procedures after the physician’ examination, in physician’s presence, and based on consultation with the physician (11).

**Figure 1:**
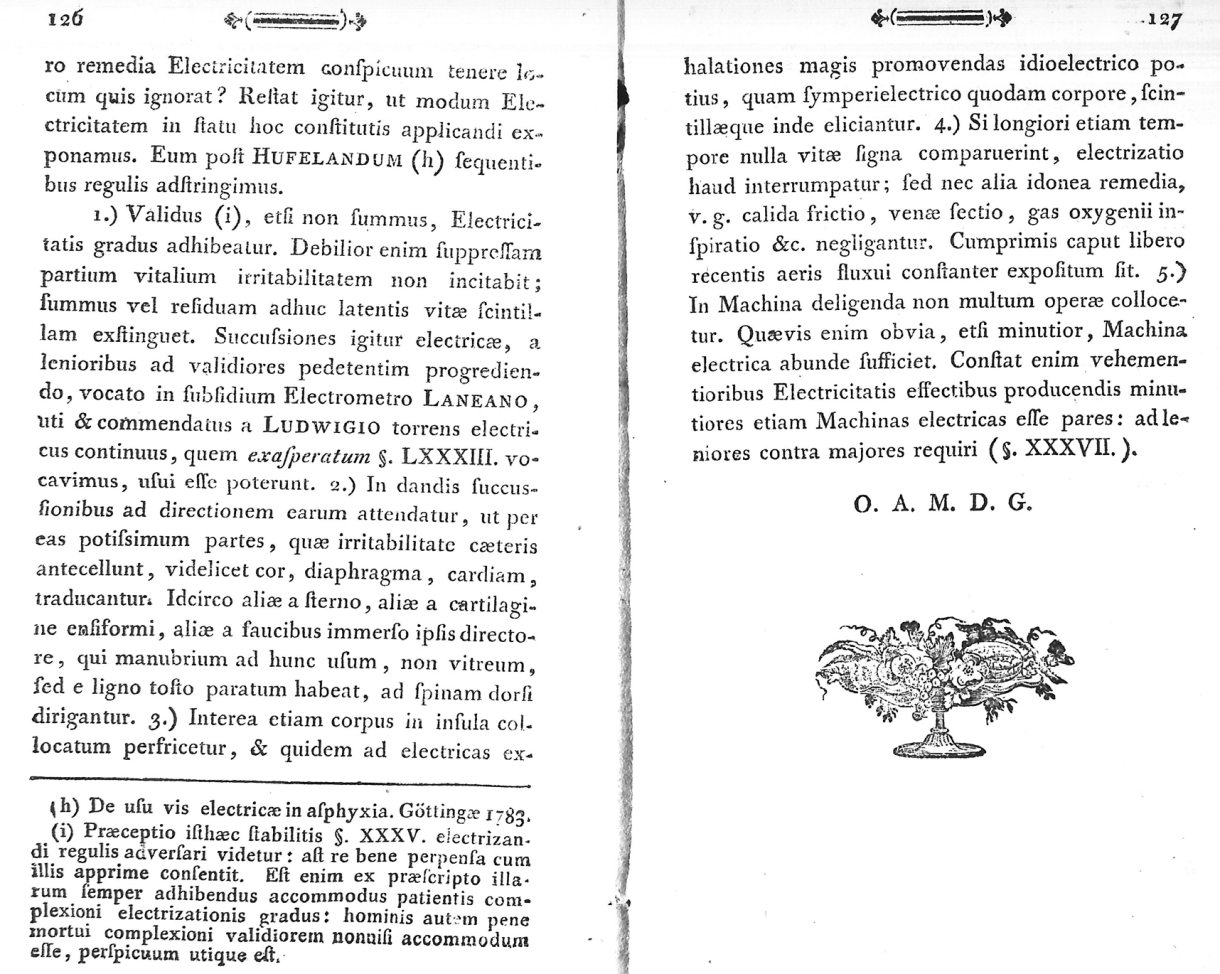
Domin’s original text describing the term “asphyxia.”

We translated Domin’s original text on resuscitation with an electric shock (“sparks”) in an apparently dead patient in whom death resulted from the cessation of circulation and life functions (*asphyxia*). In traditional Ancient medicine, the term *asphyxia (*gr. ἀσφυξία) is defined as a “pulseless condition,” but the term *mors apparens* signifies apparent death or *vita minima, vita reducta.* Domin proposes a gradual release of electricity, adjusted based on the patient’s weight, followed by a permanent electrification. In addition to placing three electrodes electrifying the area of the cardia, heart, and mouth, he emphasizes the necessity of applying parallel methods of resuscitation, such as oxygen application or inhalation (*gas oxigenii*), cold rubbing, and venesection, but does not mention specific clinical cases of resuscitation (Figure 1) (11). Here, we provide the translation of the original text:

“Asphyxia is the cessation (failure) of the arterial pulse and other life, natural and mental functions; it therefore shows apparent death. However, to raise those who for any reason fall into this pernicious state, and not to miss anything that could be attempted, a sensible hope for any help or the very sense of humanity appears to be more than enough. Therefore, it follows that electricity should and can be leant on them, since it can rightfully be expected that it could improve this pernicious condition.

Namely, if there is any appropriate remedy to repair this deadly condition, that should be the remedy that truly stimulates. Who does not know that electricity holds a noticeable place among stimulating remedies? It remains, therefore, to expound the method of applying the electricity at this state by points. According to Hufeland (footnote h: De usu vis electricae in asphyxia. Göttingen, 1783), it is bound by the following rules:

1. It is important, though not most important, to determine the level of the electricity (footnote i: the level of the electricity should be adjusted according to the patient’s weight). Namely, the weaker [electricity] will not trigger the stopped irritation of the life parts; the greatest will suppress the remaining spark of the hidden life. Therefore, releasing electric currents step by step from weaker to stronger with the help of the Laneano electrometer, and finally the recommended constant electric flow of Ludwig, which we have called exasperated in the paragraph LXXXIII, could be useful.

2. As regards the currents to be administered, care must be taken to direct them in such a way that they should best be conducted through the parts which exceed others by sensitivity: the heart, diaphragm, stomach (cardia). Therefore, they are guided to the ridge of the back in one immersed direction from the sternum, another from the pectoral muscle, and one from the mouth itself, and hereby a handle not made of glass but made of dry wood has to be used.

3. However, the body placed “on the island” should also be exposed to electrical radiations (exhalations) that are more triggered by self-electricity rather than by some electrical conductor; sparks should then be triggered.

4. If no signs of life are shown for a longer period, the electrification should not be aborted, as well as the other appropriate remedies, such as cold rubbing, venesection, oxygen application (inhalation), etc. should not be ignored. Firstly, the head should be constantly exposed to fresh air.

5. The selected machine should not be much exposed to work. Namely, any electric machine at hand, though insignificant, will be too powerful. It is certain that smaller electric machines are more suitable for producing stronger effects of electricity: smaller against more powerful ones are sought (§ XXXVII).”

## DISCUSSION

Domin acted at a turbulent intersection of the animistic and mechanistic philosophy of medicine and the still-surviving humoral theory. His contemporaries taught that electricity, as a mechanistic external agent, stimulates the body juices and the animistic part of the human body. The very name *reanimation* (resuscitation) comes from the Latin word for the return of the “animus,” spirit or life force, into the body, which was the basis of medical philosophy at the time of humoral theory until the mid-19th century (1,4,13). Electrostimulation in some cases rapidly triggered the reduced cardiac activity, that is, bradycardia/tachycardia or fibrillation, and consequently produced the fantastic result of the revival, or reanimation.

In his paper on medical history, Schmitz-Cliever describes historical resuscitation procedures including ventilation, external cardiac massage, and the development of indirect and direct defibrillation since the second half of the 19th century (2). The first experimental defibrillations, on a dog, were performed in 1899 by Prevos and Batelly. They first caused fibrillation, by inducing small electric shocks, and afterwards defibrillation, by repeating the shocks (1). A resuscitation of an apparently dead infant by an electric shock was described by Israel in his 1927 paper (14). Hyman and Hyman in 1933 performed an electric resuscitation by injecting a needle into the heart (the so-called *Hyman Otor*). External defibrillation was first performed in 1930 by Kouwenhoven, on a dog. In 1947, the surgeon Beck performed a direct defibrillation on a 14-year-old boy, who underwent a cardiac arrest due to congenital chest anomaly (15,16). As for the medical publications in Croatia, in 1886 Fon described the faradization with high current induced as a method of resuscitation after a cardiac arrest while inducing chloroform anesthesia (17). External defibrillation did not take hold until the 1970s, when it replaced direct cardiac massage with thoracotomy (18).

Lüderitz's history of electrotherapy with references to its use in resuscitation does not refer to Domin’s work. Lüderitz described a syncope recorded by Mercuriale in 1580, Morgagni’s association of syncope with bradycardia from 1761, the famous Galvani’s experiment with frogs and electroreceptivity of the muscles from 1791, the experiments of Bishat and Aldini in 1800 and 1804, as well as other experiments from the second half of the 19th century. Lüderitz also mentioned the resuscitation of a three-year-old girl in 1774 by a pharmacist who used an electrical shock lasting 20 minutes in order to perform the external electrical stimulation of the heart. This episode was also mentioned by Naumann d'Alnoncourt in 1983, and is the first description of external electrical heart stimulation (19). Hufeland, a practitioner, naturopath, macrobiotic, and anti-mesmerist from Weimar, described the effects of electrical stimulation on dead and apparently dead animals by electrifying the phrenicus (1783). He supported the theory of irritability and “*vital force*.” According to him, irritability was a consequence of poor health and should be stimulated with electricity. He referred to the condition of poor stimulation of irritability as *asthenia*. Hufeland’s work on electrical stimulation of the phrenicus in animal resuscitation was the first work on electric resuscitation in the history of medicine, although not applicable to humans. Hufeland’s work is also mentioned by Domin in his books (8,20). An overview of the use of electrotherapy in home care and hospitals, mostly for pain and paralysis, is also given by Nicolaus, who does not mention Domin’s publications on electrotherapy (21); neither does Saxtorph in 1804 (22). However, Domin was referred to by Zemplen as a meritorious historical figure for his research and applied biophysical work (23).

Although not a physician but a philosopher and physicist, Domin used his physico-chemical research for implementing the treatment procedures described in his publications upon the indication of physicians (7,12,13). Along the necessity of resuscitation, Domin also emphasizes the need for humanity on the occasion of death. This attitude was a part of his belief as an animist and a practitioner of the Enlightened intellectualism of the late 18th century.
